# Polysaccharides and Peptides With Wound Healing Activity From Bacteria and Fungi

**DOI:** 10.1002/jobm.202400510

**Published:** 2024-10-16

**Authors:** Nazli Pinar Arslan, Tugba Orak, Aysenur Ozdemir, Ramazan Altun, Nevzat Esim, Elvan Eroglu, Sinem Ilayda Karaagac, Cigdem Aktas, Mesut Taskin

**Affiliations:** ^1^ Vocational School of Health Services Bingol University Bingol Turkey; ^2^ Department of Molecular Biology and Genetics, Science Faculty Ataturk University Erzurum Turkey; ^3^ Department of Molecular Biology and Genetics, Science and Art Faculty Bingol University Bingol Turkey

**Keywords:** bacteria, fungi, peptide, polysaccharide, wound healing

## Abstract

Bacteria and fungi are natural sources of metabolites exhibiting diverse bioactive properties such as wound healing, antioxidative, antibacterial, antifungal, anti‐inflammatory, antidiabetic, and anticancer activities. Two important groups of bacteria or fungi‐derived metabolites with wound‐healing potential are polysaccharides and peptides. In addition to bacteria‐derived cellulose and hyaluronic acid and fungi‐derived chitin and chitosan, these organisms also produce different polysaccharides (e.g., exopolysaccharides) with wound‐healing potential. The most commonly used bacterial peptides in wound healing studies are bacteriocins and lipopeptides. Bacteria or fungi‐derived polysaccharides and peptides exhibit both the in vitro and the in vivo wound healing potency. In the in vivo models, including animals and humans, these metabolites positively affect wound healing by inhibiting pathogens, exhibiting antioxidant activity, modulating inflammatory response, moisturizing the wound environment, promoting the proliferation and migration of fibroblasts and keratinocytes, increasing collagen synthesis, re‐epithelialization, and angiogenesis. Therefore, peptides and polysaccharides derived from bacteria and fungi have medicinal importance. This study aims to overview current literature knowledge (especially within the past 5 years) on the in vitro and in vivo wound repair potentials of polysaccharides and peptides obtained from bacteria (Actinobacteria, Bacteroidetes, Cyanobacteria, Firmicutes, and Proteobacteria) and fungi (yeasts, filamentous microfungi, and mushrooms).

**Abbreviations**
ABTS: 2,2′‐azino‐bis(3‐ethylbenzothiazoline‐6‐sulfonic acid) diammonium salt
BC: bacterial cellulose
CPSs: cell‐bound exopolysaccharides
DPPH: 1,1‐diphenyl‐2‐picrylhydrazyl
ECM: extracellular matrix
EPSs: exopolysaccharides
GSH: glutathione
HA: hyaluronic acid
HaCaT: human epidermal keratinocyts
HDF: human dermal fibroblast
HIF‐1α: hypoxia‐inducible factor‐1α
HPLC: high‐performance liquid chromatography
HUVECs: human umbilical vein endothelial cells
IPSs: intracellular polysaccharides
MDA: malondialdehyde
NF‐κB: nuclear factor‐κB
O_2_^–^
: superoxide radical
OH: hydroxyl radical
PVA: polyvinyl alcohol
ROS: reactive oxygen species
VEGF: vascular endothelial growth factor

## 1 Introduction

Skin, the largest organ in the human body, participates in various critical processes such as thermal regulation and protection against chemicals and pathogens, as well as hydration, vitamin D synthesis, excretion, and absorption [1, 2]. Skin wounds can arise from physical, chemical, thermal, microbial, or immunological insults [3, 4]. Surgery, injuries, and burns, or pathologic conditions (diabetes or vascular diseases) are the important reasons for wounds [5].

Wound healing is a biological process consisting of four main physiological stages (hemostasis, inflammation, proliferation, and tissue remodeling) that ensures the re‐establishment of tissue integrity in the wound area [6]. Acute wounds can have their structural integrity at the end of four stages, whereas chronic wounds cannot successfully complete these four stages and, therefore, cannot be repaired regularly and in a timely manner to ensure anatomical and functional integrity even after 3 months [7, 8]. All wound types possess the potency to become chronic, and various factors (venous or arterial insufficiency, diabetes mellitus, local pressure effects, obesity, smoking, poor nutrition, infection, altered immunological status, etc.) can contribute to the conversion of acute wounds to chronic ones [7, 8, 9].

Since skin wounds, especially large acute wounds and chronic wounds reduce the quality of life and even can threaten human health, their treatment is very important for human health. There are different treatment options for the healing of wounds, such as wound dressings, natural compounds, skin substitutes, and nanoparticle application [10, 11].

The natural compounds with wound healing activity can be obtained from plants, animals, algae, fungi, bacteria, and lichens [11, 12, 13, 14, 15, 16, 17]. Polysaccharides and peptides are two examples of natural metabolites with wound‐healing activity derived from these organisms. To date, several research articles have been conducted on the wound‐healing potential of bacteria‐derived polysaccharides, especially bacterial cellulose (BC) and hyaluronic acid (HA). Even, there are some review articles on wound healing activity of bacteria‐derived other polysaccharides in addition to cellulose and HA [18, 19, 20, 21].

Bacteria are able to synthesize various peptides with wound‐healing activity, such as bacteriocins, lipopeptides, and so forth. So far, numerous research articles have been published on wound healing activities of these peptides [22, 23, 24, 25, 26, 27, 28, 29]. Furthermore, some review articles have been published on wound healing potency of bacteriocins and lipopeptides; however, in these review articles, the other biological activities (antimicrobial, anticancer, etc.) of the peptides were mostly mentioned and their wound healing potential was superficially touched upon [30, 31, 32, 33, 34, 35, 36, 37]. In short, the mentioned review articles focused on either only the wound‐healing potency of the bacterial peptides or the wound‐healing potency of bacterial polysaccharides. There are no review articles focusing on the wound‐healing activity of both bacterial peptides and bacterial polysaccharides.

Polysaccharides synthesized from fungi (yeasts, filamentous microfungi, and mushrooms) include chitin, chitosan, glucan, and mannans [38, 39, 40, 41, 42]. In the literature, the wound‐healing abilities of either chitin and chitosan or mushroom and yeast polysaccharides have been mentioned in some review articles [43, 44, 45, 46, 47]. On the contrary, there is no detailed review study in the literature regarding the wound‐healing activity of polysaccharides obtained from filamentous microfungi. Therefore, this study also focused on evaluating the current literature on polysaccharides with wound‐healing activity derived from not only yeasts and mushrooms but also filamentous fungi. Furthermore, there is no review article in the literature on the wound‐healing activity of fungal peptides. Accordingly, this work aims to integrate the current literature knowledge about polysaccharides and peptides with wound‐healing activity derived from bacteria and fungi.

The literature search for this study was conducted in scientific research platforms like Web of Science, Google Scholar, Wiley, Elsevier, Taylor and Francis, BMC Springer, and MDPI. To conduct the search, the key terms “polysaccharide,” “glucan,” “mannan,” “chitosan,” “peptide,” “bacteriocins,” “lipopeptides,” “bacteria,” “*Streptomyces*,” “lactic acid bacteria,” “Cyanobacteria,” “fungi,” “yeast,” “mushroom,” “filamentous microfungi,” or “molds” was combined with another key term “wound healing” or “wound dressing.”

## 2 Skin Wounds and Their Treatments

Skin, which is the largest organ of the human body and covers the outer surface of the body, functions as the first barrier to prevent different damaging factors (dehydration, chemical, radiological, and physical agents) and microbial attacks [48, 49]. It is composed of mainly three layers: epidermis, dermis, and hypodermis (subcutaneous layer). Epidermis, the outer layer of the skin is made up of mainly keratinocytes (about 90%–95% of epidermal cells). This layer functions as the main barrier against external environmental factors. The dermis consists mainly of a collagen‐rich extracellular matrix (ECM) where various cells are located. Fibroblasts constitute the majority of the cell density of the dermis and synthesize elastin and collagen giving strength and flexibility to the skin. The dermis also contains mast cells, macrophages, sebaceous and sweat glands, hair follicles, smooth muscle cells, blood vessels, peripheral nerves, and nerve endings [50, 51]. The hypodermis is the deepest layer of the skin and consists of fat. This layer plays a role in thermal insulation and mechanical protection of the body [52].

A wound refers to the disruption of cellular, anatomical, and functional integrity of a tissue in an organ such as skin. Physical, chemical, thermal, microbial, or immunological insults may cause the formation of a wound [3, 4]. Surgery, injuries, and burns, or pathologic conditions such as diabetes or vascular diseases are the important reasons for wounds [5]. For example, burn wounds are among the most devastating wounds, and their treatment requires long‐term treatments and numerous surgical procedures [53]. Wounds can be classified into two main categories, namely acute and chronic wounds. Acute wounds possess normal wound healing stages, which provide the regaining of restoration of anatomical and functional integrity at an appropriate time. Whereas a chronic wound exhibits an impaired healing process (a persistent inflammation phase), can not regain optimum anatomical and functional integrity, and does not heal within 3 months or longer (several months) [2, 54, 55].

Chronic wounds (leg ulcers, foot ulcers, pressure sores, etc.) result from a disordered arterial supply, a disordered venous drainage, and some metabolic diseases such as diabetes mellitus. Chronic ulceration may also be worsened by obesity, advanced age, smoking, malnutrition, diseases (e.g., AIDS), and medications (chemotherapy or radiation therapy‐related immunosuppression). Pressure or decubitus ulcers arise from constant external skin pressure, most often on the buttocks, sacrum, and heels [4, 9].

Wound repair is a biological process that mainly occurs in four stages, namely hemostasis, inflammation, proliferation (cellular infiltration, angiogenesis, and re‐epithelialization), and maturation/remodeling. When the order of these stages is disrupted, for example, when persistent inflammation occurs, wound healing stops, and the wound begins to become chronic [2, 5, 56]. Neutrophils, monocytes/macrophages, fibroblasts, endothelial cells, and keratinocytes, as well as ECM components (collagens, fibrin/fibrinogen, fibronectin, glycosaminoglycans, vitronectin, etc.) participate in the wound repair process [57, 58]. In response to injury, the first immediate response occurs in the hemostasis stage, in which collagen provokes the activation and aggregation of platelets and thus causes the accumulation of a fibrin clot at the injury zone. The inflammation stage appears immediately following the injury and lasts nearly 4–6 days. During the inflammatory phase of wound repair, the activation of immune cells causes the secretion of pro‐inflammatory cytokines that affect the migration of fibroblasts, epithelial, and endothelial cells. Simultaneously, the fragments released after the degradation of collagens induce the proliferation of fibroblasts and the synthesis of growth factors that cause angiogenesis and re‐epithelialization [52, 59]. The last stage of the wound repair process, the remodeling stage, appears simultaneously with the formation of granulation tissue. The main purpose of this stage is to provide the formation of new epithelium and scar tissue, and its completion can last a year or more [60].

There are some important factors affecting the course of the wound healing process: infection, low oxygen, inflammation level, poor blood supply, old age, nutrition, moisture level, high glucose levels, and so forth. For example, the contamination of the wound zone by pathogenic bacteria is the main problem in the wound‐healing process. Both gram‐positive bacteria and gram‐negative bacteria can lead to infections in a wound zone. Especially, long‐term chronic wounds contain dominantly *Pseudomonas*, *Acinetobacter*, and *Stenotrophomonas* [4, 61]. These bacteria cause the reduction of growth factors and the degradation of fibrins which contributes to wound healing. Furthermore, the formation of bacterial biofilm layer on chronic wounds contributes to the formation of the chronic wound environment and decreases the effectiveness of antibiotics and immune cells [4, 62].

The two main stimulants of inflammation are injury and infection. Inflammatory cells (neutrophils and macrophages) release enzymes such as collagenases and elastases to remove foreign particles and tissue debris from the wound zone. Furthermore, they generate reactive oxygen species (ROS) and antimicrobial molecules (cathepsins, defensins, lactoferrin, and lysozyme) to destroy pathogenic microorganisms [52, 63]. Although adequate levels of the inflammation process are necessary for wound healing, its excessive levels may impair the healing process and thus induce scar formation. Chronic inflammation also occurs in nonhealing wounds linked with some diseases such as diabetes and vascular disorders. This persistent inflammation can lead to abnormal degradation of the ECM and excessive breakdown of tissue, hindering the progression of healing [64]. For example, inflammation‐induced ROS have a positive effect on the wound healing process when present at adequate concentrations; however, ROS in excessive concentrations could be devastating for wound repair since it enhances oxidative stress, lipid peroxidation, and cell damage [52, 65].

The other important factors for efficient wound healing are oxygen concentration, moisture level, and nutrition deficiency. Oxygen is essential for different biological processes, such as cell proliferation, angiogenesis, and protein synthesis, which are needed for the restoration of tissue function and integrity. Therefore, providing sufficient oxygen to the wound site stimulates healing responses and positively affects the results of other treatment approaches [66]. Whereas, hypoxia interrupts fibroblast proliferation and collagen production and increases the growth of anaerobic pathogens, thereby hindering wound repair [67, 68]. Moisture levels can positively or negatively affect wound healing. Although adequate levels of moisture promote wound healing by accelerating the inflammatory and proliferative phases, excess moisture may cause maceration of the surrounding skin [69, 70]. Nutrition deficiency is another factor restricting the normal processes of wound healing. It can cause undesired effects on wound healing by prolonging the inflammatory phase, reducing fibroblast proliferation, and shifting collagen synthesis. Malnourished patients are prone to the development of pressure ulcers, infections, and chronic nonhealing wounds [71]. In brief, it can be said that preventing pathogenic bacteria, providing enough blood flow, getting enough food as well as keeping the inflammation, ROS, and moisture at adequate levels have critical importance for effective wound healing.

## 3 Natural Products for Wound Healing Treatments

Simple or acute wounds can be healed by the body's own natural mechanisms. Whereas, some treatments are needed to accelerate the healing of acute wounds or to heal difficult wounds (large wounds, chronic wounds, diabetic wounds). Wound dressings, natural products, skin substitutes, exogenous growth factors, negative pressure therapy, oxygen‐ozone therapy, shock wave therapy, photobiomodulation, and nanoparticle application are examples of therapeutic approaches used in wound healing. These treatment options are used alone or in combination for wound repair [10, 72, 73].

Wound dressings are the widest materials used for the treatment of wounds. An ideal wound dressing provides a moist environment, increases epidermal migration, promotes angiogenesis and connective tissue synthesis, permits gas exchange between injured tissue and the environment, sustains the favorable temperature for the improvement of blood flow to the wound bed, prevents bacterial infection and promotes debridement action for migration of leukocytes. Furthermore, it should be sterile, nontoxic, nonallergenic, cost‐effective, and not be stuck to the wound, and its removal should be easy [74]. In wound dressing applications, various materials, including gauze, films, plasters, bandages, cotton wool, hydrocolloids, hydrogels, and skin substitutes, are employed to provide a favorable wound moisturizing environment [75]. Furthermore, antimicrobial agents (antibiotics, nanoparticles, and silver) can be added to wound dressings to prevent pathogenic bacteria [76, 77].

The use of natural products is another option for wound healing treatments. Natural products can support the wound healing process via diverse mechanisms such as anti‐inflammatory, antioxidant, and antimicrobial activities, as well as promoting cell proliferation and pro‐collagen synthesis [78, 79]. To date, natural products such as polysaccharides, peptides, essential oils, alkaloids, flavonoids, terpenoids, saponins, and phenolic compounds have been shown to be used in wound healing treatments. These natural products with wound‐healing activity can be obtained from plants, algae, fungi, and bacteria [16, 17, 80, 81, 82].

## 4 Bioactive Metabolites From Bacteria and Fungi

The term “prokaryotic” is used to define the cells which have a nuclear material without a nuclear membrane. In other words, the genetic material of prokaryotic cells is present in a region in the cytoplasm known as the nucleoid and is not surrounded by a membrane. Their genetic information is coded in a double‐stranded circular molecule of DNA. Some prokaryotic organisms also possess small circular plasmids containing additional DNA. Furthermore, prokaryotic organisms lack membrane‐bound other organelles within their cytoplasm. The only organelle in prokaryotic organisms is the ribosome, the membrane‐less organelle where protein synthesis occurs. Prokaryotic organisms are divided into two different domains, bacteria and archaea. The members of the domain bacteria are also known as eubacteria or true bacteria [83, 84, 85].

This domain includes numerous phyla; however, some phyla, such as Actinobacteria, Bacteroidetes (Bacteroidota), Cyanobacteria, Firmicutes (Bacillota), and Proteobacteria (Pseudomonadota), are known to be more important in terms of industrial, biotechnological, and medicinal purposes. For example, bacteria are capable of producing diverse natural metabolites such as polysaccharides, pigments, enzymes, peptides, phenolics, alkaloids, terpenoids, fatty acids, amino acids, organic acids, and vitamins, which find use in food, cosmetics, textiles, and health industries [86, 87].

Fungi are eukaryotic organisms distributed in the terrestrial and aquatic environments. The kingdom of fungi consists of three major groups: single‐celled yeasts, multicellular filamentous micro‐fungi, and macroscopic filamentous fungi (mushrooms) [88]. Some of the fungi are pathogenic, while the majority of them are considered useful for humans. For instance, beneficial fungi are able to produce various bioactive natural metabolites such as polysaccharides, peptides, pigments, ergothioneine, ergosterol, phenolics, alkaloids, and polyphenols [88].

The production of natural metabolites from fungi or bacteria is carried out by solid‐state fermentation or submerged fermentation [89, 90, 91, 92, 93, 94, 95]. Solid‐state fermentations are done in tray, packed‐bed, rotating/stirred‐drum, fluidized‐bed, rocking‐drum, and stirred‐aerated bioreactors, while shake‐flasks or bioreactors are employed for submerged fermentations [96, 97].

In the production of natural metabolites, the use of fungi and bacteria has some advantages, such as rapid growth rate on simple and inexpensive media, easy manipulation of growth conditions, and easy modification by genetic engineering techniques. Besides, the metabolites of fungi and bacteria are mostly synthesized extracellularly, therefore, the extraction of their metabolites by these organisms is easier [88, 98, 99, 100]. Moreover, the chemical structures and bioactivities of bacterial or fungal metabolites differ from those of other organisms. For example, while chitosan is synthesized by fungi and bacteriocins by bacteria, plants cannot synthesize these substances. In short, there are many advantages to choosing fungi and bacteria in the production of bioactive metabolites.

The bioactive metabolites are extracted from the cell biomass of bacteria and fungi or their culture supernatants by using appropriate organic solvents such as ethanol, methanol, ethyl acetate, acetone, chloroform, and DMSO. In addition to solvents, mechanical and nonmechanical methods are also applied to the cells in the extraction of intracellular accumulated metabolites, thus disrupting the cell membranes and walls and allowing the solvents to penetrate the cells more easily. Culture supernatants are mainly used for the extraction of extracellularly secreted metabolites [27, 36, 47, 86, 94, 95, 96, 98, 101, 102, 103, 104].

The obtained extracts can be used directly in bioactivity studies. However, if it is desired to purify a target molecule in the extract, the extract is subjected to purification procedures in devices such as column chromatography, thin‐layer chromatography, and high‐performance liquid chromatography (HPLC). The chemical structures of the purified metabolite or metabolites are investigated with devices such as GC‐MS, LC‐MS, NMR, and FTIR [24, 36, 94, 98, 104, 105].

## 5 Bioactive Polysaccharides From Bacteria and Fungi

Polysaccharides are high molecular‐weight biopolymers consisting of monomers connected by glycosidic linkages. These biopolymers are synthesized by all living organisms, including plants, animals, fungi (filamentous micro‐fungi, mushrooms, and yeasts), bacteria, and algae (micro‐ and macro‐algae) [106, 107].

Based on the monomer composition, these polymers are categorized into two main groups. The first group includes homopolysaccharides made up of the repeating units of only one type of monomer, while the second group, namely heteropolysaccharides, is composed of the repeating units of two or more types of monomer [88, 106]. The most prevalent monomer of polysaccharides is D‐glucose. Sugars such as D‐fructose, D‐galactose, L‐galactose, D‐mannose, L‐arabinose, and D‐xylose are other common monomers of the polysaccharides. Furthermore, some of them may contain rare monomers such as D‐glucosamine, D‐galactosamine, *N*‐acetylneuraminic acid, *N*‐acetylmuramic acid, glucuronic and iduronic acids [106]. According to their electrical charge, polysaccharides may be further classified as cationic, anionic, nonionic, and hydrophobic. They can be further categorized according to their monosaccharide component types, chain lengths, and branching patterns [108, 109]. The functional properties and bioactivities of polysaccharides are strictly dependent on their molecular weight, monomer composition, branching degree, viscosity, chemical bond, solubility, and sulfate content [110, 111].

Bacterial polysaccharides are in the structure of heteropolysaccharide or homopolysaccharide, depending on their monomer composition [95, 112, 113]. According to their localization, bacterial polysaccharides can be categorized into two main groups: cytoplasmic storage polysaccharides and cell surface‐associated polysaccharides. Storage polysaccharides (e.g., glycogen, bacterial starch) serve as carbon and energy reserves against starvation conditions [114]. Surface‐associated polysaccharides can be classified into two different types according to their localization: capsular or cell‐bound exopolysaccharides (CPSs) and exopolysaccharides (EPSs). CPSs bind tightly to the cell surface and constitute a capsule around the cells, while EPSs are secreted into the ECM or loosely associated with the cell surface through electrostatic interactions [114, 115]. CPSs are synthesized by diverse bacterial pathogens (human pathogens, animal pathogens, or plant pathogens), such as *Escherichia coli*, *Salmonella enteriditis*, *Neisseria meningitidis*, *Actinobacillus pleuropneumoniae*, *Sinorhizobium meliloti*, *Staphylococcus aureus*, *Streptococcus zooepidemicus*, *Streptococcus pneumoniae*, *Erwinia stewartii*, *Erwinia amylovora*, and *Xanthomonas campestris* [115, 116, 117]. EPSs are synthesized by members of bacterial genera such as *Aeribacillus, Anoxybacillus, Brevibacillus, Geobacillus, Lactobacillus, Lactococcus, Streptococcus, Pediococcus, Leuconostoc*, and *Weisella*. The most known of EPSs are levan, inulin, and dextran [95, 112, 118, 119].

As with bacterial polysaccharides, fungal polysaccharides are divided into heteropolysaccharides and homopolysaccharides according to their monomer composition [120]. According to their location in the cell, fungal polysaccharides can be divided into three main categories: EPSs, cell wall polysaccharides (CWPs), and intracellular polysaccharides [121]. Chitin, chitosan, mannan, and glucan are examples of fungal CWPs [41, 122].

In fungi and bacteria, polysaccharides undertake vital functions such as energy storage, structural organization, surface adhesion, biofilm development, host colonization, virulence, and water holding as well as protecting them against cell desiccation, pathogens, host immunity, oxidizing agents, antibiotics, UV‐radiation, heavy metals and salinity [123, 124, 125, 126]. In addition to their roles in bacteria and fungi, the polysaccharides derived from bacteria or fungi are also important to humans. Humans use bacteria/fungi‐derived polysaccharides for various purposes in different industries, as well as agricultural and bioremediation studies [127, 128, 129, 130, 131, 132]. For example, bacteria/fungi‐derived polysaccharides find diverse applications in pharmaceutical, nutraceutical, medicinal, and cosmetic industries due to various biological properties including wound healing, antioxidant, antiaging, anti‐inflammatory, antidiabetic, antibacterial, antifungal, antiparasitic, antiviral, anticancer, immunomodulatory, antilipidemic, and hepatoprotective activities [88, 95, 131, 132, 133, 134, 135].

### 5.1 Polysaccharides With Wound Healing Activity From Bacteria

One of the most known polysaccharides of bacterial origin with medical or biotechnological importance is HA, which is produced in capsule form (CPS) by *S. zooepidemicus* and *Streptococcus equi*. This polysaccharide has a heteropolysaccharide structure consisting of n‐acetyl glucose amine and glucuronic acid monomers. HA is used in studies regarding cancer, osteoarthritis, plastic surgery, wound healing, drug and gene delivery, and skin and tissue engineering because of its properties such as high viscoelasticity and hydrophilicity, and binding to specific receptors as well as low immunogenicity [112, 136, 137, 138]. Furthermore, it can be used either as a component of topical formulations or as a scaffold in wound healing treatments [139, 140]. It is known that HA activates keratinocytes and participates in the proliferation, migration, and tissue maturation stages of the wound repair process [141]. For example, an in vivo study [139] elucidated that HA from *S. zooepidemicus* MTCC 3523 showed a noticeable wound‐healing activity in Wistar rats on Days 12 and 16. In a different study, Rajab, Taqa, and Al‐Wattar [79] demonstrated that when used as a component in a cream formulation, HA caused a better rate of wound healing in the skin of rabbits by inducing granulation tissue formation and re‐epithelialization. In a study, Mittal et al. [138] aimed to prepare polymeric hybrid hydrogels by using 2‐(dimethylamino) ethyl methacrylate (DMAEMA), HA, and a herbal extract of *Didymocarpus pedicellatus* (pDPi) and then to assess its wound healing potency on thickness excision wound model in rats. The first hydrogel (DMAEMA–HA) included DMAEMA and HA, while DMAEMA and HA were saturated with pDPi for the preparation of the second hydrogel (DMAEMA‐ HA‐pDPi). The results elucidated that the hydrogel DMAEMA‐HA‐pDPi provided a better wound closure rate when compared to the marketed formulation and the first hydrogel DMAEMA‐HA. The histopathologic examinations revealed that both hydrogels provided an enhanced cutaneous wound repair by promoting fibroblast proliferation, keratinocyte differentiation, re‐epithelization, granulation tissue formation, and angiogenesis.

BC is a polysaccharide that is synthesized extracellularly by the members of diverse bacterial genera, including G*luconacetobacter*, *Acetobacter*, *Agrobacterium*, *Achromobacter*, *Azobacter*, *Rhizobium*, *Alcaligenes* [142]. BC is a biocompatible polymer that does not show toxic or carcinogenic properties. BC exhibits properties during the wound healing process, such as retaining moisture, absorbing exudates from injured tissue, and accelerating granulation. Additionally, it has a nanofibril network morphology and thus can mimic the ECM [15, 143]. These properties make BC useful for diverse biomedical purposes, such as drug delivery, bone and cartilage tissue engineering, wound dressing, and skin repair. For instance, BC is added to wound dressing so that it controls wound exudates and makes an adequate moisture environment. However, it is known that BC has no antibacterial effectiveness and it is therefore combined with antibacterial agents for wound dressing application [143]. To date, some studies have been performed to elucidate its potential in the wound‐healing process (Table 1). For example, Moraes et al. [144] intended to prepare a hydrogen (BC/COL) by combining collagen (COL) with BC (from *Gluconacetobacter hansenii* ATCC 23769) and then to evaluate the efficacy of the hydrogel on wound healing in rats. BC/COL hydrogel showed a high adhesive property, provided a humid environment, and caused rapid re‐epithelialization, thereby exhibiting a good wound healing efficacy. Song et al. [145] focused on preparing a hydrogel consisting of polyvinyl alcohol (PVA), BC, and nano‐silver (AgNPs) and then investigating its usability as a wound‐healing agent in mice models. The results revealed that the PVA/BC/Ag hydrogel possessed high mechanical property and exhibited outstanding antibacterial property and good biocompatibility. Furthermore, the hydrogel was determined to promote wound healing in mice models and to provide a wound closure of 97.89% within 15 days.

**Table 1:** Bacterial polysaccharides with wound healing activity.

Compound/reference	Bacterium–Phylum	Formulation	Activities
Hyaluronic acid (capsular hetero‐polysaccharide) [79]	*Streptococcus zooepidemicus* (Firmicutes)	A cream component	Granulation tissue formation and re‐epithelialization (in vivo)
Hyaluronic acid [135]	*Streptococcus equi* (Firmicutes)	A hydrogel component	Fibroblast proliferation, keratinocyte differentiation, re‐epithelization, granulation tissue formation, and angiogenesis (in vivo)
Cellulose (extracellular) [140]	*Gluconacetobacter hansenii* (*Proteobacteria*)	A hydrogel component (Cellulose + collagen)	Showing a high adhesive property as well as providing a humid environment and causing rapid re‐epithelialization (in vivo)
Cellulose [141]	*Acetobacter xylinum* (*Proteobacteria*)	A hydrogel (polyvinyl alcohol, bacterial cellulose, and nano‐silver)	Antibacterial property and good biocompatibility (in vivo)
EPS‐S3 (exopolysaccharide) [142]	*Pantoea* sp. YU16‐S3 (Actinobacteria)	EPS‐S3 alone or as a component of ointment	Enhancement of cell adhesion, cell proliferation and cell migration, the activation of macrophages, re‐epithelialization (in vitro and in vivo)
R‐PS18 (exopolysaccharide) [143]	*Rhizobium* sp. PRIM‐18 (Proteobacteria)	R‐PS18 alone	Iron chelation and superoxide scavenging abilities, HDF cell proliferation (in vitro)
EPS‐Ca_6_ (exopolysaccharide) [144]	*Lactobacillus* sp. Ca_6_ (Firmicutes)	EPS‐Ca_6_ alone	DPPH radical scavenging, reducing power, lipid peroxidation inhibition, metal chelating, and antibacterial activity (in vitro) as well as blood vessel formation, re‐epithelialization, increased synthesis and organization of collagen fibers (in vivo)
Exopolysaccharide [132]	*Nostoc* sp. strains PCC7936 and PCC7413 (Cyanobacteria)	Hydrogel	Fibroblast migration and proliferation (in vitro)
Exopolysaccharide (FucoPol) [145]	*Enterobacter* A47 (Proteobacteria)	FucoPol or FucoPol/AgNP composite	Keratinocyte migration and antibacterial activity (in vitro)
EI6‐EPS (exopolysaccharide) [146]	*Lactiplantibacillus plantarum* (Firmicutes)	EI6‐EPS alone	Proliferation and migration of skin fibroblasts (in vitro)
Exopolysaccharide [147]	*L. plantarum* (Firmicutes)	A cream component	DPPH radical scavenging, ferric reducing power, and chelating activities (in vitro) as well as hyalinization and fibroblast proliferation (in vivo)
Levan (exopolysaccharide) [47]	*Bacillus subtilis* (Firmicutes)	A cream component	Antibacterial and antibiofilm activities (in vitro) as well as connective tissue formation and collagen production (in vivo)
Exopolysaccharide [131]	*Alteromonas* sp. PRIM‐28 (Proteobacteria)	EPS alone	Proliferation and migration of dermal fibroblasts and keratinocytes (in vitro)
EPS22 (exopolysaccharide) [148]	*Pseudomonas stutzeri* AS22 (Proteobacteria)	EPS22 alone as a hydrogel	DPPH‐radical scavenging activity as well as metal reducing and chelating abilities (in vitro) as well as neovascularization, the deposition of proteoglycans and collagens, granulation tissue formation, re‐epithelialization and faster keratinization (in vivo)
Exopolysaccharide [149]	*Bacillus licheniformis* PASS26 (Firmicutes)	EPS alone	Proliferation of human skin keratinocytes (in vitro)
Exopolysaccharide [150]	*Polaribacter* sp. SM1127 (Bacteroidetes)	A single component in phosphate‐buffered saline	Migration of HDF (in vitro) as well as fibroblast proliferation, granulation tissue formation and re‐epithelialization (in vivo)

Abbreviations: ABTS, 2,2′‐azino‐bis(3‐ethylbenzothiazoline‐6‐sulfonic acid; DPPH, 2,2‐diphenyl‐1‐picrylhydrazyl; H_2_O_2_, hydrogen peroxide; HDF, human dermal fibroblast; O_2_^•−^, superoxide radical; OH^•^, hydroxyl radical.

In addition to HA and cellulose, bacteria also produce other polysaccharides with wound healing activity. For instance, Sahana and Rekha [146] focused on characterizing an exopolysaccharide (EPS‐S3) from a marine bacterium *Pantoea* sp. YU16‐S3 and then evaluating for its wound‐healing potential in vitro and in vivo. The structural analysis displayed that EPS‐S3 possesses a molecular weight of 1.75 × 10^5^ Da and is a heteropolysaccharide consisting of glucose, galactose, N‐acetyl galactosamine, and glucosamine. EPS‐S3 displayed cell adhesion and proliferation potential, facilitated the migration of fibroblasts, and also supported the activation of macrophages. The in vivo experiments exhibited that EPS‐S3 caused the re‐epithelialization of damaged tissue by increasing the expression of HB‐EGF, FGF, E‐cadherin, and β‐catenin through the Wnt/β‐catenin pathway. A different research team [147] intended to examine the structural properties and bio‐active properties (in vitro antioxidant, cell proliferative, and wound healing capacities) of EPS (R‐PS18) from *Rhizobium* sp. PRIM‐18. The EPS was determined to be made up of glucose, galactose, and mannose, exhibit iron chelation and superoxide scavenging abilities, and significantly increase human dermal fibroblast (HDF) cell proliferation and wound healing. In a previous study, Trabelsi et al. [148] focused on evaluating antibacterial and antioxidant properties (in vitro) and wound healing potency (in vivo) of a novel EPS (EPS‐Ca_6_) from *Lactobacillus* sp. Ca_6_ strain. The researchers found that EPS‐Ca_6_ possessed antioxidative property according to DPPH radical scavenging, reducing power, lipid peroxidation inhibition, and metal chelating activity assays. They also found that EPS‐Ca_6_ displayed noticeable antibacterial towards against *Salmonella enterica* and *Micrococcus luteus*. In the excision wound model of rats, it was determined that EPS‐Ca_6_ caused blood vessel formation and re‐epithelialization, provided deposition and an organization of collagen fibers (remodeling phase), and increased hydroxyproline levels, thereby exhibiting a good wound healing potential. In a study performed by Alvarez et al. [149], the EPSs obtained from two different strains (PCC7936 and PCC7413) of *Nostoc* were characterized, and then the usability of EPSs as a biomaterial in wound dressing application was assessed. The structural analysis verified the anionic nature and the presence of sulfate groups and uronic acids in both EPS. The in vitro scratch assay indicated that the hydrogels prepared from EPSs were biocompatible and had the potency to enhance fibroblast migration and proliferation. Overall, the study showed that the *Nostoc* EPSs have the potential to heal skin injuries. In a study conducted by Concordio‐Reis et al. [150], a fucose‐containing polysaccharide (FucoPol) from the bacterium *Enterobacter* A47 was obtained, and AgNP was synthesized, FucoPol/AgNP biocomposite was prepared, and then the biocomposite was tested for wound dressings application. Both FucoPol and FucoPol/AgNP biocomposite supported the in vitro keratinocyte migration but displayed no cytotoxicity on human skin keratinocytes and mouse fibroblasts. Furthermore, FucoPol/AgNP exhibited powerful antimicrobial effectiveness towards two skin commensal pathogens, namely *S. aureus* ATCC 6538 and *Klebsiella pneumoniae* CECT 8453. Overall, the study indicated that the FucoPol has the potential to be used for the preparation of wound healing formulations. Zaghloul and Ibrahim [151] aimed to investigate the wound‐healing activity of the EPS from a marine isolate *Lactiplantibacillus plantarum* EI6. The EPS was determined to consist of different monomers (rhamnose, galactose, mannose, glucose, and arabinose) and induce human skin fibroblast proliferation and migration. Elmansy et al. [152] aimed to characterize the EPS from *L. plantarum* RO30 and then test its antioxidant and wound‐healing activities. The research team found that the EPS has a molecular weight of 4.96 × 10^4^ g/mol and was a heteropolysaccharide consisting of glucuronic acid, mannose, glucose, and arabinose monomers. They also reported that EPS had an in vitro antioxidative capacity according to the DPPH radical scavenging, reducing power, and iron chelation activity assays. Furthermore, they revealed that EPS possessed wound‐healing potential on burn wound models in rats. In a study performed by Hamada et al. [47], it was determined that the levan from *Bacillus subtilis* MZ292983.1 exhibits antibacterial effectiveness towards two pathogenic bacteria (*S. aureus* and *E. coli*) and the antibiofilm property. Moreover, the study revealed that the levan accelerated the healing of burn‐induced wounds in rats by increasing connective tissue, collagen production, and organization. Sahana and Rekha [153] aimed to investigate the structural properties and wound‐healing ability of EPS from *Alteromonas* sp. PRIM‐28. The EPS was determined to be an anionic heteropolysaccharide with a molecular weight of 780 kDa, which consists of mannuronic acid, glucose, and N‐acetyl glucosamine monomers and includes sulfate, phosphate, and uronic acid residues. EPS displayed biocompatibility and supported the proliferation and migration of dermal fibroblasts and keratinocytes. In a previous study, Maalej et al. [154] aimed to investigate the in vitro antioxidative activity and the in vivo wound healing potency of EPS from *Pseudomonas stutzeri* AS22. The in vitro antioxidant activity assay displayed that the EPS possessed DPPH‐radical scavenging activity as well as metal‐reducing and chelating abilities. The in vivo animal experiments elucidated that when EPS was applied on dermal full‐thickness excision wounds in rats, it could enhance wound healing and provide total closure within 12 days. Histological findings revealed that EPS caused the formation of a well‐organized layer in both derma and epidermis (strong re‐epithelialization and keratinization, neovascularization, and the deposition of proteoglycans and collagens), meaning it provides tissue regeneration and wound healing. A different research team [155] focused on examining the structural properties and biological activities of the EPS from *Bacillus licheniformis* PASS26. The EPS was determined to be a heteropolysaccharide with a low molecular weight of 56 kDa consisting of glucose, galactose, fructose, mannose, and galacturonic acid. The experiments regarding biological activity revealed that EPS had both the in vitro antitumor activity and the in vitro wound healing potential. In a study performed by Sun et al. [156], the researchers evaluated the skin wound healing and frostbite injury prevention potentials of EPS from the Arctic marine bacterium *Polaribacter* sp. SM1127. The in vitro assay revealed that the EPS promoted the migration of HDFs. The in vivo experiments elucidated that when applied to full‐thickness cutaneous wounds in rats, EPS accelerated the wound healing rate by increasing fibroblast proliferation, granulation tissue formation, and re‐epithelialization.

### 5.2 Polysaccharides With Wound Healing Activity From Fungi

Chitin and chitosan are the most known examples of polysaccharides with wound‐healing activity synthesized by fungi. These natural biopolymers with a polysaccharide structure can be synthesized by fungi in addition to crustaceans and insects [157, 158]. The fungal classes of *Basidiomycetes, Ascomycetes*, *Zygomycetes*, and *Deuteromycetes* are reported to comprise chitin and chitosan in their cell walls [159]. Chitin and chitosan possess various biological properties, including antitumor, antimicrobial, and antioxidant activities; however, chitosan is more serviceable for biotechnological and medicinal purposes since it has higher solubility, biological activity, and reactivity with other substances [160]. Especially some biological properties (low toxicity, biodegradability, hemostatic potency and antimicrobial effectiveness) make chitosan and its derivatives more useful for wound dressing applications [52]. To date, the wound healing potential of crustaceans or insects‐derived chitosan has been documented in numerous studies. Similarly, chitin/chitosan‐containing mycelia of fungi or fungi‐derived pure chitin/chitosan have been tested as wound healing material in several studies (Table 2) [161, 162, 163, 164, 165, 169]. For example, Yasrebi et al. [164] examined the wound‐healing potency of chitosan from a mushroom *Trametes versicolor*. For this, chitosan and PVA (chitosan/PVA) nanofibers were prepared by electrospinning method, and then their antibacterial, cytotoxic and wound healing activities were tested. The fibers (chitosan/PVA) were determined to exhibit antibacterial efficiency towards two pathogens (*E. coli* and *S. aureus*) and also increase the adhesion and growth of fibroblasts by facilitating the exchange of moisture and oxygen. Animal experiments elucidated that the chitosan/PVA fibers provided a wound‐healing potential of 95%. In a previous study, Chen et al. [165] aimed to prepare the chitosan (rhizochitosan) from the chitin (rhizochitin) which was extracted from *Rhizopus stolonifer* F6, and then to assess the wound healing potency of rhizochitosan. The experiments elucidated that when applied on rats with full‐thickness injury, the composite dressing consisting of rhizochitosan and Regenplex exhibited a stronger healing potency as compared to each individual component and control group. The composite was considered to enhance wound healing by affecting inflammation and remodeling stages. The action mechanism of the composite on wound healing was ascribed to the reduction of the level of matrix metalloproteinase (MMP)‐9 expression in the early stage and the enhancement of MMP‐2 expression level in a later stage of the healing process.

**Table 2:** Fungal polysaccharides with wound healing activity.

Compound	Bacterium–Phylum	Formulation	Activities
Chitosan [161]	*Trametes versicolor* (mushroom)	Chitosan/PVA nanofibers	Antibacterial efficiency (in vitro) as well as enhancement of adhesion and growth of fibroblasts (in vivo)
Chitosan [162]	*Rhizopus stolonifer* F6 (filamentous microfungus)	Composite (chitosan + Regenplex)	Release of growth factors, the induction of hair follicle development, the regulation of inflammation and remodeling stages as well as the reduction of the level of MMP‐9 expression in early stage and the enhancement of MMP‐2 expression level in a later stage of healing process in vivo)
LEP‐2b (Exopolysaccharide) [151]	*Lachnum* YM405 (filamentous microfungus)	A component of ointment	Enhancement of re‐epithelialization and hydroxyproline levels as well as antioxidative and immunoregulatory activities (in vivo)
TEPS1 and TEPS2 (Exopolysaccharides) [163]	*Talaromyces purpureogenus* (filamentous microfungus)	TEPS1 alone or TEPS2 alone	In vitro antioxidant property (DPPH, ABTS, and •OH radical scavenging activities) and wound healing activities (in vitro)
PNPs (PNP‐40, PNP‐60, and PNP‐80) (Polysaccharides) [152]	*Pholiota nameko* (mushroom)	Each PNP alone	Antioxidant (hydroxyl radical scavenging activity and intracellular ROS scavenging) and anti‐collagenase activities as well as fibroblast proliferation and migration (in vitro)
β‐glucan (polysaccharide) [(164)]	*Lignosus rhinoceroti* (mushroom)	β‐glucan alone	The migration and proliferation of intestinal epithelial cells, as well as the enhancement of the expression levels of cell division control proteins 42, Rac‐1, RhoA, and Par‐3 (in vitro)
Polysaccharide [153]	*Phellinus igniarius* (mushroom)	A hydrogel containing chitosan and polysaccharide	Antioxidant and antibacterial activities (in vitro) as well as re‐epithelialization, collagen deposition, reduced inflammation, and enhanced angiogenesis (in vivo)
Polysaccharide [165]	*Agaricus blazei* (mushroom)	Films (Polysaccharide/sodium alginate/polyvinyl alcohol)	Reduction in oxidative stress markers (MDA and nitrite/nitrate levels) and the increase in collagen deposition (*In vivo)*
Scleroglucan (Scl) and EPS‐R (Exopolysaccharides) [42]	*Sclerotium glucanicum* (filamentous microfungus) and *Rhodosporidium babjevae* (yeast)	Scl alone or EPS‐R alone	Macrophages and fibroblasts migration, collagen biosynthesis (in vivo)
Exopolysaccharide [166]	*Papiliotrema terrestris* (yeast)	Exopolysaccharide alone	Fibroblast proliferation, and antibacterial and antioxidant activities (in vitro) as well collagen fibers synthesis and maturation, hair follicle development, and re‐epithelialization (in vivo)
Chitosan‐glucan [167]	*Schizophyllum commune* (mushroom)	A component of wound dressing	Enhancement of cell adhesion, the absorption of excessive exudates, and antibacterial effectiveness (in vitro) as well as the enhancement of angiogenesis, re‐epithelization, granulation, and cell migration (in vivo)
Chitin‐glucan [168]	*S. commune* (mushroom)	Nanofibers prepared from chitin‐glucan, poly(vinyl alcohol), and gelatine	Antibacterial effectiveness, the proliferation and adhesion of fibroblasts (in vitro and in vivo)
Beta‐glucans [38]	*S. commune* (mushroom)	Beta‐glucans dissolved in butylene glycol	Keratinocyte migration, dermal fibroblast differentiation and proliferation as well as re‐epithelialization (in vitro and in vivo)
Exopolysaccharide [40]	*Rhodosporidium mucilaginosa* (yeast)	Polysaccharide, polycaprolactone, and gelatin	The proliferation of endothelial cells and fibroblasts as well as the enhancement of angiogenesis (in vivo)
β‐D‐glucan (lasiodiplodan) [39]	*Lasiodiplodia theobromae* (filamentous microfungus)	A hydrogel	Antioxidant activity (in vitro) as well as immunomodulatory effect, collagen synthesis, and cell re‐epithelialization and proliferation (in vivo)

Abbreviations: ABTS, 2,2′‐azino‐bis(3‐ethylbenzothiazoline‐6‐sulfonic acid; DPPH, 2,2‐diphenyl‐1‐picrylhydrazyl; H_2_O_2_, hydrogen peroxide; HDF, human dermal fibroblast; MMP‐2, matrix metalloproteinase‐2; MMP‐9, matrix metalloproteinase‐9; O_2_^•−^, superoxide radical; OH^•^, hydroxyl radical.

In addition to chitin/chitosan, fungi‐derived other polysaccharides have also been reported to wound healing activity. For example, He et al. [166] aimed to characterize an exopolysaccharide (LEP‐2b) from the filamentous microfungus *Lachnum* YM405 and then to investigate the in vivo wound healing potency of LEP‐2b in mice. The structural and biochemical analysis revealed that LEP‐2b is a heteropolysaccharide with a molecular weight of 2.8 × 10^4^ Da, which consists of rhamnose, mannose, glucose, and galactose monomers. LEP‐2b application caused rapid decrustation of the wounded skin, decreased the healing time and also augmented the water and hydroxyproline levels in the repaired skin. Overall, it was determined that this EPS inhibited the inflammatory reaction of scalded skin, accelerated tissue repair, and re‐epithelialization, and thus supported wound healing. In another study performed recently [167], the structural properties and wound healing potential of two EPS (TEPS1 and TEPS2) from endophytic fungus *Talaromyces purpureogenus* were investigated. HPLC‐based monomer analysis revealed that TEPS1 was a heteropolysaccharide consisting of mannose, ribose, glucose, and galactose whereas TEPS2 was a homopolysaccharide made up of only mannose. The in vitro antioxidant analysis displayed that TEPS1 was determined to have a higher antioxidant activity than TEPS2 based on DPPH, ABTS, and •OH radical scavenging assays. Furthermore, TEPS1 was proven to have higher cellular antioxidant and wound‐healing activities in the human embryonic kidney (HEK293) cell line. In a study, Sung et al. [168] aimed to examine the in vitro antioxidative, anti‐collagenase, and wound healing properties of the polysaccharides extracted from an edible mushroom *Pholiota nameko*. The experiments revealed that the polysaccharides, especially the PNP‐80 fraction, exhibited a hydroxyl radical scavenging activity and anti‐collagenase activity and caused a noticeable decrease in cellular ROS content of H_2_O_2_‐induced L929 cells. Furthermore, PNP‐80 was determined to notably boost the proliferation and migration of L929 fibroblasts, indicating its potent wound‐healing activity. A different research group [170] focused on characterizing the polysaccharide isolated from a medicinal mushroom *Lignosus rhinocerotis*, and then examining its intestinal mucosal wound healing activity. The isolated polysaccharide was identified as β‐glucan and its molecular weight was determined as 5.315 × 10^4^ g/mol. This β‐glucan was found to induce migration and proliferation of intestinal epithelial cells by activating the Rho‐dependent pathway. Based on these results, the investigators suggested that β‐glucan can be utilized as a wound‐healing material for the treatment of diseases with gastrointestinal mucosal damage. Zhang et al. [171] aimed to prepare a hydrogel (PCA) based on L‐arginine conjugated chitosan and aldehyde functionalized polysaccharides of a mushroom *Phellinus igniarius* and then to investigate the potential usability of PCA as antibacterial and pro‐angiogenesis dressing material in the wound healing process of diabetic rats. The researchers determined that PCA possessed good antioxidant, antibacterial, and biological safety and exhibited an effective wound‐healing potential in diabetic rats. Histological analysis proved that PCA supported the epithelial formation and collagen deposition, and protein expression analysis supported that PCA reduced the inflammation via the inhibition of nuclear factor‐kappa B inhibitor alpha (IKBα)/nuclear factor‐κB (NF‐κB) signaling pathway and increased angiogenesis via the adjustment of the level of hypoxia‐inducible factor‐1α (HIF‐1α). In another study performed recently, Saraiva et al. [172] focused on isolating the polysaccharides (PAbs) from *Agaricus blazei* Murill mushroom, preparing the films (PAbs/SA/PVA) based on sodium alginate and PVA loaded with PAbs and then investigating the potential of PAbs/SA/PVA in the healing of cutaneous wounds in mice. The experiments elucidated that the films promoted the formation of a thicker dermis with greater collagen deposition and caused a noticeable decrease in oxidative stress markers (malondialdehyde [MDA] and nitrite/nitrate levels). Hamidi et al. [42] aimed to investigate the in vitro bioactive properties and the in vivo wound healing abilities of EPSs (scleroglucan and EPS‐R, respectively) obtained from a filamentous microfungus *Sclerotium glucanicum* DSM 2159 and a yeast *Rhodosporidium babjevae*. The in vitro assays revealed that both EPSs were noncytotoxic on the human fibroblast cell line and did not cause a noticeable hemolysis or detriment to human red blood cells (hemocompatibility). The in vivo tests revealed that both EPSs displayed a favorable wound‐healing potential when applied to male Wistar rats at a concentration of 10 mg/mL. Hamidi et al. [173] aimed to investigate the structural properties of the EPS from the yeast *Papiliotrema terrestris* PT22AV and then evaluate its antibacterial, cytocompatibility, and wound healing properties. The structural and biochemical analysis showed that the EPS was a water‐soluble heteropolysaccharide with an average molecular weight of 202 kDa, which consisted of mannose and glucose monomers. The in vitro tests elucidated that the EPS possessed antimicrobial potency against the pathogenic bacteria (*E. coli*, *S. aureus*, and *Staphylococcus epidermidis*) and exhibited cytocompatibility towards human fibroblast and macrophage cell lines. The in vivo experiments displayed that the EPS exhibited wound healing potency by increasing collagen fibers synthesis and maturation, hair follicle development, and re‐epithelialization, especially when applied to male rats at a concentration of 10 mg/mL. In a study performed by Abdel‐Mohsen et al. [174], the toxicity and biological activities (antibacterial and wound healing) of the chitin/chitosan‐glucan complex isolated from a mushroom *Schizophyllum commune* were investigated. These researchers found that the complex exhibited strong antibacterial efficiency against different pathogenic bacteria but had no cytotoxicity against mouse fibroblast cells. They also informed that the complex possessed excellent surgical wound healing potential on rat models. In previous work, Zeynali et al. [175] aimed to prepare the chitin‐glucan complex (CGC)/PVA/Gelatin nanofibers from the CGC (from *S. commune*), PVA, and gelatine by using the electrospinning process, and then to investigate the toxicity profile and biological activities (antibacterial and wound healing potential) of CGC/PVA/Gelatin nanofibers. The experiments revealed that CGC/PVA/Gelatin nanofibers possessed an inhibition effect against *E. coli* and *S. aureus*, did not cause toxic effects on fibroblasts, and also improved proliferation and adhesion of fibroblasts. Furthermore, CGC/PVA/Gelatin nanofibers provided a wound healing potential of 86% when applied on grade 2 burn wounds in rats. In a study performed by Seo et al. [38], beta‐glucans from mushrooms (*S. commune*), barley, yeast, and euglena were tested for their wound‐healing potential. The in vitro analysis revealed that all the beta‐glucans possessed a notable influence on keratinocyte migration at 20 μM and exhibited no cytotoxicity on fibroblasts. The in vivo experiments concluded that the beta‐glucan derived from mushroom‐induced keratinocyte migration via the induction of FAK/Src phosphorylation and activated dermal fibroblast differentiation through NADPH oxidase, thereby accelerating wound closure. In the work carried out by Hivechi et al. [40], the EPS from cold‐adapted yeast *Rhodosporidium mucilaginosa* sp. GUMS16 was evaluated for its effect on the healing of full‐thickness wounds in rats. For this, the nanofibers that were prepared from the purified EPS, polycaprolactone (PCL), and gelatin were applied to wounds. EPS‐based formulations (PCL/Gel/1% EPS and PCL/Gel/2% EPS) provided the highest wound closure efficiency and lowest scar signs and caused more synthesis of endothelial cells and fibroblasts in the wound area. Furthermore, EPS‐based formulations were determined to accelerate angiogenesis and support the formation of dermal appendices (hair follicles and sweat glands) when compared to PCL/Gel and control groups. In a different study [39], the researchers aimed to prepare the hydrogel based on the exocellular (1 → 6)‐β‐D‐glucan (lasiodiplodan) derived from a filamentous microfungus *Lasiodiplodia theobromae* and then to investigate the physico‐chemical properties and wound healing activity of the prepared hydrogel. The results revealed that the hydrogel has a suitable pH for topical application and physicochemical stability and exhibits antioxidant potential to scavenge hydroxyl radicals. The in vivo experiments elucidated that the hydrogel exhibited immunomodulatory activity and promoted cell re‐epithelialization and proliferation as well as the synthesis of collagen fibers, thereby providing a strong wound healing potential.

## 6 Bioactive Peptides From Bacteria and Fungi

Bioactive peptides are specific amino acid sequences that are generated mostly from plant and animal proteins by enzymatic hydrolysis, acid‐alkaline hydrolysis, and microbial fermentation. Furthermore, they are also generated from the protein‐rich biomass of algae, yeast, and fungi using appropriate hydrolysis methods. On the contrary, some bioactive peptides can also be produced naturally by bacteria, archaea, fungi, algae, animals, and plants [88].

Bacteria‐derived natural peptides can be classified into two main groups according to their biosynthesis: ribosomally synthesized peptides and nonribosomally synthesized peptides. Ribosomally synthesized bacterial peptides contain a great deal of chemical, structural, and functional diversity [176]. The second group, namely nonribosomally synthesized ones, are not encoded by a gene and are synthesized by nonribosomal peptide synthases (NRPS). Unlike ribosomal synthesis, which uses messenger RNA as the template for the amino acid sequence of the synthesized peptide, NRPS delivers one amino acid to the growing peptide chain [177].

Fungi‐derived peptides are mainly extracted from the fermented culture media of the fungi using appropriate solvents, most commonly ethyl acetate. Then, the prepared extracts were subjected to evaporation under vacuum at 40°C until dryness, thus obtaining a semi‐solid residue [178]. Fungal peptides are produced naturally by different fungal genera, including *Acremonium, Alternaria, Aspergillus, Asteromyces, Beauveria, Chaetomium, Eurotium, Gliocladium, Graphium, Metarrhizium, Penicillium, Simplicillium, Stagonospora, Talaromyces*, and *Trichoderma* [179, 180]. In the case of bacterial peptides, the fungal peptides are also synthesized ribosomally or nonribosomally (by NRPS) [181, 182]. The fungi‐derived peptides can be grouped into three main categories: linear (linear‐dipeptides, tripeptides, tetra‐ and hexapeptides, octapeptides, lipopeptides, nonapeptides, undecapeptides, dodecapeptides, pentadecapeptides, etc.), cyclic (dipeptides, tripeptides, tetrapeptides, pentapeptides, hexapeptides, heptapeptides, nonapeptides, decapeptides, etc.), and depsipeptides (cyclic depsipeptides and cyclic tripeptides) [178, 183].

Bacteria/fungi‐derived peptides exhibit diverse health‐beneficial biological properties, such as antimicrobial (antibacterial and antifungal), antioxidant, anti‐inflammatory, wound healing, antiviral, antiparasitic, anticancer, antiobesity, anti‐adipogenic, anti‐hypertensive, antithrombotic, and antidiabetic activities [178, 183].

### 6.1 Peptides With Wound Healing Activity From Bacteria

Bacteriocins are examples of ribosomally synthesized bacterial peptides in lengths of 20–60 amino acids. They are synthesized by both gram‐negative and gram‐positive bacteria. Lactic acid bacteria such as *Lactobacillus*, *Lactococcus*, *Streptococcus*, *Enterococcus*, and *Pediococcus* species are the most known examples of bacteriocins‐producing bacteria. Bacteriocins can be divided into three groups according to their structure and properties: class I, class II, and class III [184, 185]. Class I, also named lantibiotics are posttranscriptionally modified bacteriocins with small size (< 5 kDa), which typically comprise 19–50 amino acids. A widespread property of this group is the presence of unusual amino acids, including dehydrated amino acids, lanthionine, and 3‐methyllanthionine. Nisin and lactocin are examples of Class I bacteriocins [76, 185]. Class II bacteriocins include small thermostable (< 10 kDa) peptides with an amphiphilic helical structure such as plantaricin, pediocin, lactococcin, and sakacin. They do not possess posttranslational modifications in their peptide chains. They are categorized into three subclasses: subclass II‐A, subclass II‐B, and subclass II‐C. Class III bacteriocins include large heat‐stable bacteriocins with a molecular weight of more than 30 kDa. Helveticin J, enterolysin, and millericin B are examples of class III bacteriocins [186, 187].

Bacteriocins exhibit strong antimicrobial activity, and therefore they are used for the preparation of drug formulation and as food preservatives [188, 189]. They are also used as wound‐healing agents due to their antibacterial efficiency (Table 3). For instance, in a study [23], the antibacterial and wound‐healing properties of the enterocin from *Enterococcus faecalis* were investigated. The enterocin was found to be efficient towards the pathogen bacteria (*S. aureus*, *K. pneumoniae*, *Enterobacter cloaca*, *Listeria monocytogenes*, and *Proteus vulgaris*). In addition, the experiments revealed that the enterocin improved wound healing process by increasing epithelialization and granulation tissue formation. In a different study, Ovchinnikov et al. [190] aimed to characterize an antimicrobial substance from *Staphylococcus equorum* and then investigate its antimicrobial effectiveness in a murine skin wound infection model. The antimicrobial metabolite was identified as the thiopeptide bacteriocin micrococcin P1 (MP1). This bacteriocin was found to possess antimicrobial effectiveness towards many gram‐positive bacteria, including methicillin‐resistant *S. aureus* (MRSA), especially when combined with rifampicin. The combination (MP1‐rifampicin) was able to eradicate and prevent the recurrence of Xen31 (multidrug‐resistant luciferase‐tagged MRSA strain Xen31) infection on wound. A different research team [24] intended to investigate the wound‐healing potency of Nisin A from *Lactococcus lactis*. The experiments revealed that the Nisin A enhanced notably migration of both human umbilical vein endothelial cells (HUVECs) and human epidermal keratinocytes (HaCaT), provided re‐epithelization of the porcine skin, and inhibited *E. coli* growth. Overall, the research team suggested the usage of Nisin A as a wound‐healing agent, since it augmented the mobility of skin cells, dampened the influence of lipopolysaccharide and pro‐inflammatory cytokines, and reduced bacterial growth. A study undertaken by Preet, Kaur, and Raza [191] focused on developing a nisin‐loaded carbopol gel formulation (NLCG) and then exploring its therapeutic efficiency against *Pseudomonas aeruginosa* infected burn wounds. The researchers found that NLCG significantly inhibited *Pseudomonas* in the skin but had no in vitro cytotoxicity on erythrocytes and peritoneal macrophages. Based on the results from percentage wound closure, tensile strength, histological, and scanning electron microscopic studies, NLCG was determined to increase the restoration of the skin epithelium and exhibit a wound healing activity. NLCG was also proven to cause a noticeable modulation in hydroxyproline content, myeloperoxidase levels, and serum levels of IL‐1, IL‐10, and TNF‐α. Overall, the researchers reported that NLCG had anti‐*Pseudomonas*, wound healing, and immunomodulatory effectiveness. In a different work by Preet et al. [192], the influence of nisin on the healing of streptozotocin‐induced diabetic wounds in rats was investigated. The experiments revealed that nisin treatment increased collagen content and skin regeneration as well as the restoration of different layers of skin tissue. Nisin treatment was also determined to reduce the oxidative stress in the wound microenvironment by increasing the levels of antioxidants and scavenging free radicals.

**Table 3:** Bacteria‐derived peptides with wound healing activity.

Compound	Bacterium–Phylum	Formulation	Activities
Enterocin (ribosomally synthesized bacteriocin) [23]	*Enterococcus faecalis* (Proteobacteria)	Enterocin alone or Enterocin + Eusol	Antibacterial efficiency (in vitro) as well as epithelialization and granulation tissue formation (in vivo)
Micrococcin P1 (Bacteriocin) [184]	*Staphylococcus equorum* (Firmicutes)	MP1 + rifampicin	Antibacterial efficiency on wound (in vivo)
Nisin (ribosomally synthesized bacteriocin) [24]	*Lactococcus lactis* (Firmicutes)	Nisin alone	Migration of human umbilical vein endothelial cell and HaCaT cells (in vivo) as well as re‐epithelization (ex vivo)
Nisin (ribosomally synthesized bacteriocin) [185]		A nisin‐loaded carbopol gel formulation (NLCG)	Antibacterial efficiency, immunomodulatory effect, tensile strength, re‐epithelization as well the increase in hydroxyproline content (in vivo)
Nisin (ribosomally synthesized bacteriocin) [186]		Nisin alone	The enhancement of collagen content and skin regeneration as well as the enhancement of antioxidant activity (in vivo)
Surfactin A (nonribosomally synthesized lipopeptide) [25]	*Bacillus subtilis* (Firmicutes)		Keratinocyte migration, the inhibition of scar tissue formation, the upregulation of HIF‐1α and VEGF, as well as the regulation of pro‐inflammatory cytokines levels (*In vivo*)
Lipopeptides (nonribosomally synthesized) [22]	*Bacillus mojavensis* A21 (Firmicutes)	Lipopeptide‐based hydrogel	Antioxidant activity (DPPH radical‐scavenging, reducing power, and lipid peroxidation inhibition properties) (in vitro) as well as epidermal regeneration and re‐epithelialization (in vivo)
Lipopeptide (nonribosomally synthesized) [190]	*B. subtilis* SPB1 (Firmicutes)	Lipopeptide‐based gel	Antioxidant activity (DPPH‐scavenging, reducing power, lipid peroxidation inhibition, and iron chelating potentials) (in vitro) as well epidermal regeneration and complete re‐epithelialization (in vivo)
Bacillomycin D (nonribosomally synthesized lipopeptide) [189]		Bacillomycin + amphotericin	Antibiofilm activity and keratinocyte cell migration (in vitro)
Lipopeptide (nonribosomally synthesized) [26]	*Acinetobacter junii* (Proteobacteria)	The formulation containing lipopeptide, carbomer, polyethylene glycol, and ethanolamine	Complete epidermization, granulation tissue formation, collagen deposition, and mild inflammatory cell infiltration (in vivo)
Lipopeptide (nonribosomally synthesized) [191]	*A. junii* B6	A component of topical gel	DPPH radical scavenging and ferric reducing power potential (in vitro) as well as the reduction of oxidative stress (MDA, H_2_O_2_, and GSH levels), lesion size, neutrophilic inflammation, erythema, and edema, and the enhancement of re‐epithelialization and hair follicle development (in vivo)
Lipopeptide (nonribosomally synthesized) [192]	*A. junii* B6	Lipopeptide alone	Migration and tube formation of HUVECs as well as the enhancement in the expression level of angiogenic‐related genes (HIF‐1α and VEGF) (in vitro)
Lipopeptides (nonribosomally synthesized) [27]	*Halomonas venusta* (Proteobacteria)		Prevention of adhesion and biofilm‐forming potential of pathogens and the enhancement of cell proliferation and migration activities (in vitro) as well as fibroblast and macrophage migration, dense connective tissue formation, collagen regeneration, and epithelialization (in vivo)
Daptomycin (nonribosomally synthesized lipopeptide) [193]	*Streptomyces roseosporus* (Actinobacteria)	Daptomycin alone	Antibacterial effectiveness, epithelialization, and collagen synthesis (in vivo)
ε‐polylysine (nonribosomally synthesized) [28]	*Streptomyces* sp. (Actinobacteria)	Hydrogel (ε‐polylysine, alginic acid, dopamine, and acrylamide)	Antimicrobial effectiveness, reduction of inflammation, enhancement of angiogenesis, and collagen deposition (in vivo)
Actinomycin X2 [29]	*Streptomyces cyaneofuscatus* (Actinobacteria)	Film (actinomycin X2 and silk fibroin)	Antimicrobial effectiveness (in vitro) as well as hemocompatibility and vascularization (in vivo)

Abbreviations: DPPH, 2,2‐diphenyl‐1‐picrylhydrazyl; GSH, glutathione; H_2_O_2_, hydrogen peroxide; HaCaT, human epidermal keratinocytes; HDF, human dermal fibroblast; HIF‐1α, hypoxia‐inducible factor‐1α; HUVECs, human umbilical vein endothelial cells; MDA, malondialdehyde; VEGF, vascular endothelial growth factor.

Apart from bacteriocins, other ribosomally synthesized peptides have been documented to possess wound‐healing potential (Table 3). Lipopeptides are microbial surfactants synthesized extracellularly or as part of the cell membrane by several bacterial and fungal species. In the bacteria, their synthesis is performed in a ribosome‐independent manner by NRPS [193, 194]. *Bacillus* and *Pseudomonas* members are potential producers of lipopeptides. Examples of lipopeptides derived from *Bacillus* species are surfactins (surfactin, lichenysin, etc.), fengycins (fengycin and plipastatin), iturins (iturin A and B, bacillomycin L, mycosubtilin, etc.), antiadhesin, fusaricidin, and so forth. The most known lipopeptides produced by *Pseudomonas* species are vincosins, syringomycins, amphisins, putisolvin, tolaasin, syringopeptin, and so forth [193]. Lipopeptides are known to have strong antimicrobial effectiveness. Furthermore, they exhibit strong wound‐healing activity [22, 195]. For instance, Yan et al. [25] elucidated that surfactin A, an amphipathic cyclic lipopeptide from *B. subtilis* provided a wound closure of 80.65%. In wound healing mechanism, the peptide was found to upregulate the expression of HIF‐1α and vascular endothelial growth factor (VEGF), accelerate keratinocyte migration through mitogen‐activated protein kinase (MAPK) and NF‐κB signaling pathways, and regulate the secretion of pro‐inflammatory cytokines and macrophage phenotypic switch. Furthermore, surfactin A was proven to have a good capacity to prevent scar tissue formation by influencing the expression levels of α‐smooth muscle actin (α‐SMA) and transforming growth factor (TGF‐β). In previous research, Ayed et al. [22] aimed to assess the in vitro antioxidant activity and the in vivo wound healing potential of lipopeptides obtained by *Bacillus mojavensis* A21. The in vitro antioxidant activity assays demonstrated that the lipopeptides possessed DPPH radical‐scavenging, reducing power, and lipid peroxidation inhibition properties. When applied to the wound site in rats, the lipopeptides were determined to accelerate wound healing, provide full re‐epithelialization, and lead to the complete closure of the wound after 13 days. In a different study [196], the in vitro antioxidant activity and the in vivo wound healing capacity of *B. subtilis* SPB1 lipopeptide biosurfactant were investigated. The lipopeptide was determined to have DPPH‐scavenging activity as well as reducing power, lipid peroxidation inhibition and iron chelating potentials. The topical application of the lipopeptide‐based gel boosted notably the rate of wound closure after a period of 13 days. The histological analysis revealed that the lipopeptide provides the perfect epidermal regeneration and complete re‐epithelialization of the wound. A study undertaken by Tabbene et al. [195] assessed the potential of the lipopeptide bacillomycin D alone and the antifungal drug amphotericin B alone or their combination, to prevent *Candida albicans* biofilm formation and to support the migration of the keratinocytes. The combination was determined to be superior and support the closure of a gap made in a monolayer of human keratinocytes. A different research team [197] focused on examining the antioxidant and wound‐healing potentials of the lipopeptide obtained from *Acinetobacter junii* B6. Based on the results from DPPH radical scavenging and ferric‐reducing power assays, the researchers reported that the peptide had antioxidative potential. Similarly, the in vivo antioxidant activity assays exhibited that the peptide could reduce oxidative stress (MDA, H_2_O_2_, and GSH levels) when applied to the wounds in rats. The analyses also revealed that LBS reduced lesion size, neutrophilic inflammation, erythema, and edema but augmented re‐epithelialization and hair follicle development. Afsharipour et al. [26] aimed to prepare a nano‐lipopeptide biosurfactant (NLPB) formulation consisting of lipopeptide (from *A. junii*), carbomer, polyethylene glycol, and ethanolamine, and then investigate the usability of this formulation as an in vivo wound repair agent. The experiments indicated that NLPB hydrogels provide wound closure rates of 80% and 100%, respectively, on Days 7 and 15. This formulation was determined to provide an ulcer bed with complete epidermization, granulation tissue formation with full maturation, collagen deposition, and mild inflammatory cell infiltration. Mehrabani et al. [198] focused on examining the effect of lipopeptide obtained by *A. junii* B6 on the angiogenic potential of HUVECs. LPB led to noticeable increments in migration and tube formation of HUVECs when applied at a concentration of 300 µg/mL. Furthermore, LPB was determined to cause the expression of angiogenic‐related genes (HIF‐1α and VEGF). Overall, the authors suggested that the lipopeptide may have the potential to be used as a potential wound healing agent. Cheffi et al. [27] investigated the biosurfactant lipopeptides (Bio‐PHKT) from a halotolerant marine strain PHKT of *Halomonas venusta*. Bios‐PHKT lipopeptides were determined to have anti‐adhesive and anti‐biofilm potentials, as well as antiproliferative activity against the B16 melanoma cell line. The in vitro Scratch wound healing test revealed that Bios‐PHKT promoted cell proliferation and migration and provided notable wound closure. The in vivo experiments elucidated that when Bios‐PHKT was tested on circular excision wound models in female rats, its 5 and 10 mg/mL concentrations provided wound closure of 93.6% and 98.1% after 13 days of treatment. Bios‐PHKT was determined to promote fibroblast and macrophage migration, the formation of dense connective tissue, collagen regeneration, and great epithelialization. Overall, the Bios‐PHKT was reported to possess excellent in vitro and in vivo wound healing abilities. Simonetti et al. [199] aimed to investigate the in vivo efficacy of Daptomycin, a cyclic lipopeptide antibiotic of nonribosomal origin, in the treatment of burn wound infections induced by MRSA. They found that the daptomycin‐applied group had the highest potential to inhibit the infection. Moreover, the daptomycin‐applied group was found to have higher re‐epithelialization and collagen scores and, thus, better wound healing capacity.

There are other nonribosomally synthesized peptides with wound‐healing activity (Table 3). For example, Zhang et al. [28] aimed to prepare a novel nanocomposite hydrogel (ODPA) consisting of oxidized alginic acid, dopamine, ε‐polylysine (a nonribosomally synthesized antimicrobial peptide from *Streptomyces*), and acrylamide, and then to investigate its wound healing activity in rats. ODPA hydrogel was determined to show antimicrobial effectiveness, reduce inflammation, promote angiogenesis, and collagen deposition, thereby accelerating the healing of infected full‐thickness wounds. In a recent study [29], an antimicrobial peptide, actinomycin X2 (Ac.X2), isolated from the fermentation medium of *Streptomyces cyaneofuscatus* was immobilized onto silk fibroin (SF) fibers, and the prepared film (ASF) (Ac. X2‐immobilized SF) was then evaluated for its antibacterial and wound healing potentials. The ASF film exerted a strong antimicrobial action towards *S. aureus* and MRSA and also exhibited a suitable degradation rate, good hemocompatibility, and biocompatibility. Moreover, the film was determined to support skin regeneration and wound vascularization and restore skin function when applied to the full‐thickness wound in rats. Overall, it was suggested that the ASF film may be used as a wound dressing agent for clinical application.

### 6.2 Peptides With Wound Healing Activity From Fungi

To date, many natural peptides of fungal origin have been described in the literature, and their diverse biological properties have been reported; however, the role of fungal peptides on wound healing has been tested in only one study. In the mentioned study [200], the peptide obtained from a marine fungus *Acremonium* sp. strain CNQ‐049 was characterized and then its wound healing activity was evaluated. According to spectroscopic (MS, UV, and NMR) data analyses, the peptide was identified as a cyclic pentadepsipeptide and named acremonamide. The peptide applied to skin wounds in mice was found to exhibit wound healing activity by enhancing keratinocyte and fibroblast migration and upregulating the expression of wound healing‐related genes, namely actin alpha and collagen type I alpha.

**Figure 1:**
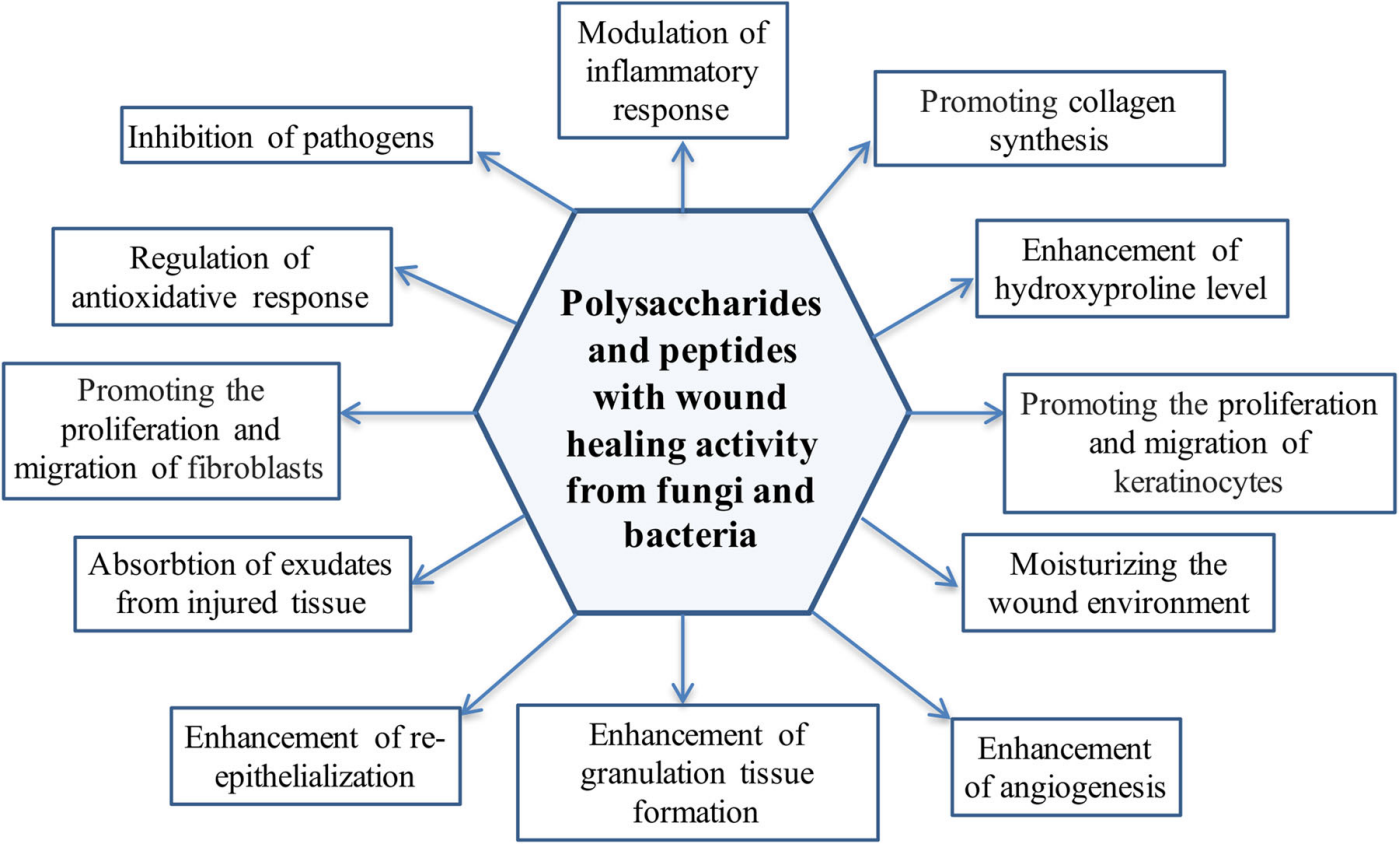
Effect mechanisms of polysaccharides and peptides from fungi and bacteria on wound healing.

In summary, the findings from previous studies show that polysaccharides and/or peptides from bacteria and fungi can facilitate wound healing by inducing proliferation and migration of fibroblasts and keratinocytes, increasing collagen synthesis, re‐epithelialization, cell adhesion and angiogenesis, activating macrophages, inhibiting pathogens, providing moisture environment, exhibiting antioxidant and anti‐inflammatory properties (Figure 1).

## 7 Conclusion and Future Perspectives

Skin wounds are an important health problem that reduces the quality of life, causes psychological problems, and can even threaten human life. Natural products obtained from organisms such as plants, animals, fungi, bacteria, and algae have the potential to be used in the treatment of skin wounds, especially large or chronic wounds. Polysaccharides and peptides are examples of natural products used in wound healing treatments. In addition to bacteria‐derived cellulose and HA and fungi‐derived chitin and chitosan, other polysaccharides from bacteria and fungi, especially their extracellular polysaccharides, exhibit in vitro and in vivo wound healing activity. Similarly, bacteria‐derived peptides, especially bacteriocins, and lipopeptides, exhibit strong wound‐healing activity. Overall, this review work indicates that polysaccharides and peptides from bacteria or fungi have wound‐healing potential. However, this review study also revealed some gaps in the literature regarding their polysaccharides or peptides. The first one is that polysaccharides of both bacteria and fungi have been less studied than those of plants for their wound‐healing potential, with the exception of cellulose and HA for bacteria and chitin and chitosan for fungi. Therefore, we recommend focusing more on bacterial and fungal polysaccharides, especially their EPSs, for the discovery of new polysaccharides with wound‐healing potential. The second is that, unlike bacteria‐derived peptides, there is only one study in the literature on the wound‐healing potential of fungi‐derived peptides. Therefore, we recommend further studies on the wound healing ability of fungal peptides.

## Author Contributions

**Nazli Pinar Arslan:** conceptualization, investigation, writing–original draft, visualization, validation, data curation. **Tugba Orak:** investigation, writing–original draft, validation. **Aysenur Ozdemir:** investigation, writing–original draft. **Ramazan Altun:** validation. **Nevzat Esim:** investigation, writing–original draft, validation. **Elvan Eroglu:** investigation, writing–original draft, validation. **Sinem Ilayda Karaagac:** investigation, writing–original draft, validation. **Cigdem Aktas:** investigation, writing–original draft, validation. **Mesut Taskin:** conceptualization, investigation, writing–original draft, data curation, validation, supervision, writing–review and editing.

## Conflicts of Interest

The authors declare no conflicts of interest.

## Data Availability

Data sharing is not applicable to this article as no new data were created or analyzed in this study.
